# Correction: Oropouche infection in Peruvian patients: A systematic review and meta-analysis

**DOI:** 10.1371/journal.pone.0350951

**Published:** 2026-06-02

**Authors:** Darwin A. León-Figueroa, Edwin Aguirre-Milachay, Milagros Diaz-Torres, Jean Pierre Villanueva-De La Cruz, Edwin A. Garcia-Vasquez, Virgilio E. Failoc-Rojas, Mario J. Valladares-Garrido

There is an error in reference 2. The correct reference is: Lorenz C, et al. Oropouche fever outbreak in Brazil: Key factors behind the largest epidemic in history. PLoS One. 2025.
