# Supplementary material for: Oropouche infection in Peruvian patients: A systematic review and meta-analysis
Source: PLoS One. 2025 Dec 4;20(12):e0337522. doi: 10.1371/journal.pone.0337522 (PMC12677477; doi:10.1371/journal.pone.0337522)
Supplement: S2 Table — (DOCX) [file pone.0337522.s002.docx]

| S2 Table. The adjusted search terms as per searched electronic databases or search tools. | | | |
| --- | --- | --- | --- |
| PubMed | | | |
|  | #1 | Oropouche[Title/Abstract] OR "Oropouche orthobunyavirus"[Title/Abstract] | 374 |
|  | #2 | Peru[Title] OR Peruvian[Title] | 9,930 |
|  | #3 | #1 AND #2 | 18 |
| Scopus | | | |
|  | #1 | TITLE-ABS ( oropouche OR "Oropouche orthobunyavirus" ) | 396 |
|  | #2 | TITLE(Peru OR Peruvian) | 36,268 |
|  | #3 | #1 AND #2 | 19 |
| Embase | | | |
|  | #1 | ('oropouche orthobunyavirus'/exp OR 'oropouche'/exp).ti,ab | 384 |
|  | #2 | (' Peru' OR 'Peruvian').ti,ab. | 48,213 |
|  | #3 | #1 AND #2 | 17 |
| Web of Science | | | |
|  | #1 | TI=(Oropouche OR “Oropouche orthobunyavirus”) | 220 |
|  | #2 | TI=(Peru OR Peruvian) | 25,537 |
|  | #3 | #1 AND #2 | 8 |
| ScienceDirect | | | |
|  | #1 | Title, abstract, keywords: Oropouche OR “Oropouche orthobunyavirus” | 118 |
|  | #2 | Title, abstract, keywords: ( Peru OR Peruvian ) | 9,638 |
|  | #3 | #1 AND #2 | 7 |
| Google Scholar | | |  |
|  | #1 | allintitle: Oropouche | 640 |
|  | #2 | allintitle: "Peru OR Peruvian" | 163,000 |
|  | #3 | #1 AND #2 | 15 |
| Virtual Health Library (VHL) | | | |
|  | #1 | ti:( Oropouche OR “Oropouche orthobunyavirus” ) | 308 |
|  | #2 | ti:( Peru OR Peruvian) | 20,796 |
|  | #3 | #1 AND #2 | 10 |
| Scielo | | | |
|  | #1 | (Oropouche) | 32 |
|  | #2 | ti:(*Peru) | 7,207 |
|  | #3 | #1 AND #2 | 4 |
| Dimensions | | | |
|  | #1 | Title and abstract: Oropouche OR “Oropouche orthobunyavirus” | 539 |
|  | #2 | ti:(*Peru) | 105,246 |
|  | #3 | #1 AND #2 | 43 |
| Epistemónikos | | | |
|  | #1 | title:(Oropouche OR "Oropouche orthobunyavirus") | 114 |
|  | #2 | ti:(*Peru) | 2,777 |
|  | #3 | #1 AND #2 | 11 |
| TOTAL |  | PubMed (n =18); Scopus (n=19); Web of Sciences(n=8); Embase (n=17); ScienceDirect (n=7); Google Scholar (n=15); Virtual Health Library (n=10); Scielo (n=4), Dimensions (n=43), and Epistemónikos (n=11) | 152 |

**Updated through April 10, 2025.**
