# Supplementary material for: Oropouche infection in Peruvian patients: A systematic review and meta-analysis
Source: PLoS One. 2025 Dec 4;20(12):e0337522. doi: 10.1371/journal.pone.0337522 (PMC12677477; doi:10.1371/journal.pone.0337522)
Supplement: S3 Table — (DOCX) [file pone.0337522.s003.docx]

**S3 Table**. Quality of the dengue studies included in the review

| Authors | Year | Eligibility criteria | Study subjects and the setting | Exposure measured in a valid and reliable way 'gold standard' | A specified diagnosis or definition | Confounding factors | Dealing with confounding factors | Outcomes measured in a valid and reliable way | Appropriate statistical analysis | Scores (8) | Quality (high, moderate, low) |  |
| --- | --- | --- | --- | --- | --- | --- | --- | --- | --- | --- | --- | --- |
| Durango-Chavez HV, et al. (1) | | 2022 | Yes | Yes | Yes | Yes | Unclear | NA | Yes | Yes | 6 | Moderate |
| Watts DM, et al. (2) | | 2022 | Yes | Yes | Yes | Yes | Unclear | NA | Yes | Yes | 6 | Moderate |
| Martins-Luna J, et al. (3) | | 2020 | Yes | Yes | Yes | Yes | Unclear | NA | Yes | Yes | 6 | Moderate |
| Silva-Caso W, et al. (4) | | 2019 | Yes | Yes | Yes | Yes | Unclear | NA | Yes | Yes | 6 | Moderate |
| Alva-Urcia C, et al. (5) | | 2017 | Yes | Yes | Yes | Yes | Unclear | NA | Yes | Yes | 6 | Moderate |
| Alvarez-Falconi P, et al. (6) | | 2010 | Yes | Yes | Yes | Yes | Unclear | NA | Yes | Yes | 6 | Moderate |

NA: Not assessed

Moola S, Munn Z, Tufanaru C, Aromataris E, Sears K, Sfetcu R, Currie M, Qureshi R, Mattis P, Lisy K, Mu P-F Chapter 7: Systematic reviews of etiology and risk: JBI; 2020. Available from: <https://synthesismanual.jbi.global>

1. Durango-Chavez HV, Toro-Huamanchumo CJ, Silva-Caso W, Martins-Luna J, Aguilar-Luis MA, Del Valle-Mendoza J, et al. Oropouche virus infection in patients with acute febrile syndrome: Is a predictive model based solely on signs and symptoms useful? PloS One. 2022;17(7):e0270294.

2. Watts DM, Russell KL, Wooster MT, Sharp TW, Morrison AC, Kochel TJ, et al. Etiologies of Acute Undifferentiated Febrile Illnesses in and near Iquitos from 1993 to 1999 in the Amazon River Basin of Peru. Am J Trop Med Hyg. 14 de noviembre de 2022;107(5):1114-28.

3. Martins-Luna J, Del Valle-Mendoza J, Silva-Caso W, Sandoval I, Del Valle LJ, Palomares-Reyes C, et al. Oropouche infection a neglected arbovirus in patients with acute febrile illness from the Peruvian coast. BMC Res Notes. 10 de febrero de 2020;13(1):67.

4. Silva-Caso W, Aguilar-Luis MA, Palomares-Reyes C, Mazulis F, Weilg C, Del Valle LJ, et al. First outbreak of Oropouche Fever reported in a non-endemic western region of the Peruvian Amazon: Molecular diagnosis and clinical characteristics. Int J Infect Dis IJID Off Publ Int Soc Infect Dis. junio de 2019;83:139-44.

5. Alva-Urcia C, Aguilar-Luis MA, Palomares-Reyes C, Silva-Caso W, Suarez-Ognio L, Weilg P, et al. Emerging and reemerging arboviruses: A new threat in Eastern Peru. PloS One. 2017;12(11):e0187897.

6. Alvarez-Falconi PP, Ruiz BAR. Brote de Fiebre de Oropuche en Bagazán, San Martín - Perú: Evaluación Epidemiológica, Manifestaciones Gastrointestinales y Hemorrágicas. Rev Gastroenterol Perú. 2010;334-40.
