# Supplementary material for: Oropouche infection in Peruvian patients: A systematic review and meta-analysis
Source: PLoS One. 2025 Dec 4;20(12):e0337522. doi: 10.1371/journal.pone.0337522 (PMC12677477; doi:10.1371/journal.pone.0337522)
Supplement: S5 Table — (DOCX) [file pone.0337522.s005.docx]

**S5 Table**. Table of excluded studies.

| **Authors** | **Year** | **Title** | **Exclusion** |
| --- | --- | --- | --- |
| **Aguilar P, et al.** [1] | 2011 | Iquitos Virus: A Novel Reassortant Orthobunyavirus Associated with Human Illness in Peru | Diseases belonging to the same genus, Orthobunyavirus. |
| **Catillo Oré R, et al.** [2] | 2018 | Molecular and antigenic characterization of group C orthobunyaviruses isolated in Peru | Diseases belonging to the same genus, Orthobunyavirus. |
| **Forshey B, et al.** [3] | 2010 | Arboviral Etiologies of Acute Febrile Illnesses in Western South America, 2000–2007 | Incorrect country |
| **Baisley K, et al.** [4] | 1998 | Epidemiology of endemic Oropouche virus transmission in upper Amazonian Peru. | Incomplete version |
| **Olortegui MP, et al.** [5] | 2024 | Genomic Epidemiology of 2023–2024 Oropouche Outbreak in Iquitos, Peru reveals independent origin from a concurrent outbreak in Brazil | Incorrect study design |
| **García M, et al.** [6] | 2016 | Detection of Oropouche viral circulation in Madre de Dios region, Peru (December 2015 to January 2016) | Incorrect study design |
| **Watts D, et al.** [7] | 1997 | Venezuelan Equine Encephalitis and Oropouche Virus Infections among Peruvian Army Troops in the Amazon Region of Peru | Incomplete version |
| **Rosa JF, et al.** [8] | 2017 | Oropouche Virus: Clinical, Epidemiological, and Molecular Aspects of a Neglected Orthobunyavirus | Incorrect study design |

[1] Aguilar PV, Barrett AD, Saeed MF, Watts DM, Russell K, Guevara C, et al. Iquitos Virus: A Novel Reassortant Orthobunyavirus Associated with Human Illness in Peru. PLoS Negl Trop Dis 2011;5:e1315. https://doi.org/10.1371/journal.pntd.0001315.

[2] Oré RMC, Caceda RE, Huaman AA, Williams M, Hang J, Juarez DE, et al. Molecular and antigenic characterization of group C orthobunyaviruses isolated in Peru. PLOS ONE 2018;13:e0200576. https://doi.org/10.1371/journal.pone.0200576.

[3] Forshey BM, Guevara C, Laguna-Torres VA, Cespedes M, Vargas J, Gianella A, et al. Arboviral Etiologies of Acute Febrile Illnesses in Western South America, 2000–2007. PLoS Negl Trop Dis 2010;4:e787. https://doi.org/10.1371/journal.pntd.0000787.

[4] Baisley KJ, Watts DM, Munstermann LE, Wilson ML. Epidemiology of endemic Oropouche virus transmission in upper Amazonian Peru. 1998. https://doi.org/10.4269/ajtmh.1998.59.710.

[5] Olortegui MP, Schiaffino F, Peñataro_Yori P, Colston JM, Shapiama_Lopez V, Pinedo_Vasquez T, et al. Genomic Epidemiology of 2023–2024 Oropouche Outbreak in Iquitos, Peru reveals independent origin from a concurrent outbreak in Brazil. medRxiv 2024:2024.12.08.24318674. https://doi.org/10.1101/2024.12.08.24318674.

[6] García MP, Merino NS, Figueroa D, Marcelo A, V ET, Manrique C, et al. Detección de la circulación del virus Oropuche en la región Madre de Dios, Perú (diciembre 2015 - enero 2016). Rev Peru Med Exp Salud Pública 2016:380–1. https://doi.org/10.17843/rpmesp.2016.332.2098.

[7] Watts DM, Lavera V, Callahan J, Rossi C, Oberste MS, Roehrig JT, et al. Venezuelan Equine Encephalitis and Oropouche Virus Infections among Peruvian Army Troops in the Amazon Region of Peru 1997. https://doi.org/10.4269/ajtmh.1997.56.661.

[8] Rosa JFT da, Souza WM de, Pinheiro F de P, Figueiredo ML, Cardoso JF, Acrani GO, et al. Oropouche Virus: Clinical, Epidemiological, and Molecular Aspects of a Neglected Orthobunyavirus 2017. https://doi.org/10.4269/ajtmh.16-0672.
