# Supplementary material for: Oropouche infection in Peruvian patients: A systematic review and meta-analysis
Source: PLoS One. 2025 Dec 4;20(12):e0337522. doi: 10.1371/journal.pone.0337522 (PMC12677477; doi:10.1371/journal.pone.0337522)
Supplement: S6 Table — (DOCX) [file pone.0337522.s006.docx]

**S6 Table**. Meta-analysis database

| **Authors** | **Year** | **Fever** | **Headache** | **Myalgia** | **Arthralgia** | **Anorexia / Hyporexia** | **Retroocular pain** | **Abdominal pain** | **Nausea / Vomiting** | **Diarrhea** | **Chills** | **Lumbar pain** | **Odynophagia** | **Cutaneous rash** | **Conjunctival injection** | **Petechiae** | **Cough** |
| --- | --- | --- | --- | --- | --- | --- | --- | --- | --- | --- | --- | --- | --- | --- | --- | --- | --- |
| **Durango-Chavez HV, et al.** [1] | 2022 | 97 | 82 | 75 | 63 | 62 | 52 | NR | NR | NR | NR | NR | 43 | 24 | NR | 1 | NR |
| **Watts DM, et al.** [2] | 2022 | 66 | 63 | NR | 56 | NR | 46 | NR | 35 | 18 | 61 | NR | NR | 10 | NR | NR | 16 |
| **Martins-Luna J, et al.** [3] | 2020 | 131 | 112 | 106 | 95 | 89 | 70 | 4 | 62 | NR | 0 | 66 | 48 | NR | 1 | 1 | 0 |
| **Silva-Caso W, et al.** [4] | 2019 | 46 | 35 | 35 | 30 | 23 | 28 | 4 | 22 | NR | NR | 16 | 16 | 15 | NR | 0 | NR |
| **Alva-Urcia C, et al.** [5] | 2017 | 12 | 8 | 6 | 7 | 4 | 4 | 0 | 3 | 1 | 3 | NR | 0 | 0 | 0 | NR | 0 |
| **Alvarez-Falconi P, et al.** [6] | 2010 | 38 | 38 | 31 | 26 | 19 | 11 | 13 | 15 | 4 | 27 | 23 | 1 | 1 | 19 | 1 | 16 |

NR: Not reported

[1] Durango-Chavez HV, Toro-Huamanchumo CJ, Silva-Caso W, Martins-Luna J, Aguilar-Luis MA, Del Valle-Mendoza J, et al. Oropouche virus infection in patients with acute febrile syndrome: Is a predictive model based solely on signs and symptoms useful? PloS One 2022;17:e0270294. https://doi.org/10.1371/journal.pone.0270294.

[2] Watts DM, Russell KL, Wooster MT, Sharp TW, Morrison AC, Kochel TJ, et al. Etiologies of Acute Undifferentiated Febrile Illnesses in and near Iquitos from 1993 to 1999 in the Amazon River Basin of Peru. Am J Trop Med Hyg 2022;107:1114–28. https://doi.org/10.4269/ajtmh.22-0259.

[3] Martins-Luna J, Del Valle-Mendoza J, Silva-Caso W, Sandoval I, Del Valle LJ, Palomares-Reyes C, et al. Oropouche infection a neglected arbovirus in patients with acute febrile illness from the Peruvian coast. BMC Res Notes 2020;13:67. https://doi.org/10.1186/s13104-020-4937-1.

[4] Silva-Caso W, Aguilar-Luis MA, Palomares-Reyes C, Mazulis F, Weilg C, Del Valle LJ, et al. First outbreak of Oropouche Fever reported in a non-endemic western region of the Peruvian Amazon: Molecular diagnosis and clinical characteristics. Int J Infect Dis IJID Off Publ Int Soc Infect Dis 2019;83:139–44. https://doi.org/10.1016/j.ijid.2019.04.011.

[5] Alva-Urcia C, Aguilar-Luis MA, Palomares-Reyes C, Silva-Caso W, Suarez-Ognio L, Weilg P, et al. Emerging and reemerging arboviruses: A new threat in Eastern Peru. PloS One 2017;12:e0187897. https://doi.org/10.1371/journal.pone.0187897.

[6] Alvarez-Falconi PP, Ruiz BAR. Brote de Fiebre de Oropuche en Bagazán, San Martín - Perú: Evaluación Epidemiológica, Manifestaciones Gastrointestinales y Hemorrágicas. Rev Gastroenterol Perú 2010:334–40. https://doi.org/10.47892/rgp.2010.304.421.
