# Supplementary material for: Oropouche infection in Peruvian patients: A systematic review and meta-analysis
Source: PLoS One. 2025 Dec 4;20(12):e0337522. doi: 10.1371/journal.pone.0337522 (PMC12677477; doi:10.1371/journal.pone.0337522)
Supplement: S7 Table — (DOCX) [file pone.0337522.s007.docx]

**S7 Table**. R version 4.2.3. script

| **Import database** |
| --- |
| Environment – From Excel – Import Excel Data - Import |
| **Calculate the prevalence of clinical characteristics** |
| library(meta)  library(readxl)  data_prevalence<- **Database name**  mtprop=metaprop(event= **Clinical characteristics**,  n= **Sample_** **Study**,  studlab=paste(Study,year),  data=data_prevalence,  method.tau = "DL",  method = "Inverse",  method.ci = "SACC",  sm="PFT")  mtprop  forest(mtprop,  comb.fixed=FALSE,  common = FALSE)  **An example is given**:  Database name: **Base_Fever**   \| Study \| year \| **Sample** \| **Fever** \| \| --- \| --- \| --- \| --- \| \| **Durango-Chavez HV, et al.** \| 2022 \| 97 \| 97 \| \| **Watts DM, et al.** \| 2022 \| 66 \| 68 \| \| **Martins-Luna J, et al.** \| 2020 \| 131 \| 131 \| \| **Silva-Caso W, et al.** \| 2019 \| 46 \| 46 \| \| **Alva-Urcia C, et al.** \| 2017 \| 12 \| 12 \| \| **Alvarez-Falconi P, et al.** \| 2010 \| 38 \| 42 \|   **Code in R version 4.2.3**  library(meta)  library(readxl)  data_prevalence<- **Base_Fever**  mtprop=metaprop(event= **Fever**,  n= **Sample**,  studlab=paste(Study,year),  data=data_prevalence,  method.tau = "DL",  method = "Inverse",  method.ci = "SACC",  sm="PFT")  mtprop  forest(mtprop,  comb.fixed=FALSE,  common = FALSE) |
