# Supplementary figures and images for: Oropouche infection in Peruvian patients: A systematic review and meta-analysis
Source: PLoS One. 2025 Dec 4;20(12):e0337522. doi: 10.1371/journal.pone.0337522 (PMC12677477; doi:10.1371/journal.pone.0337522)

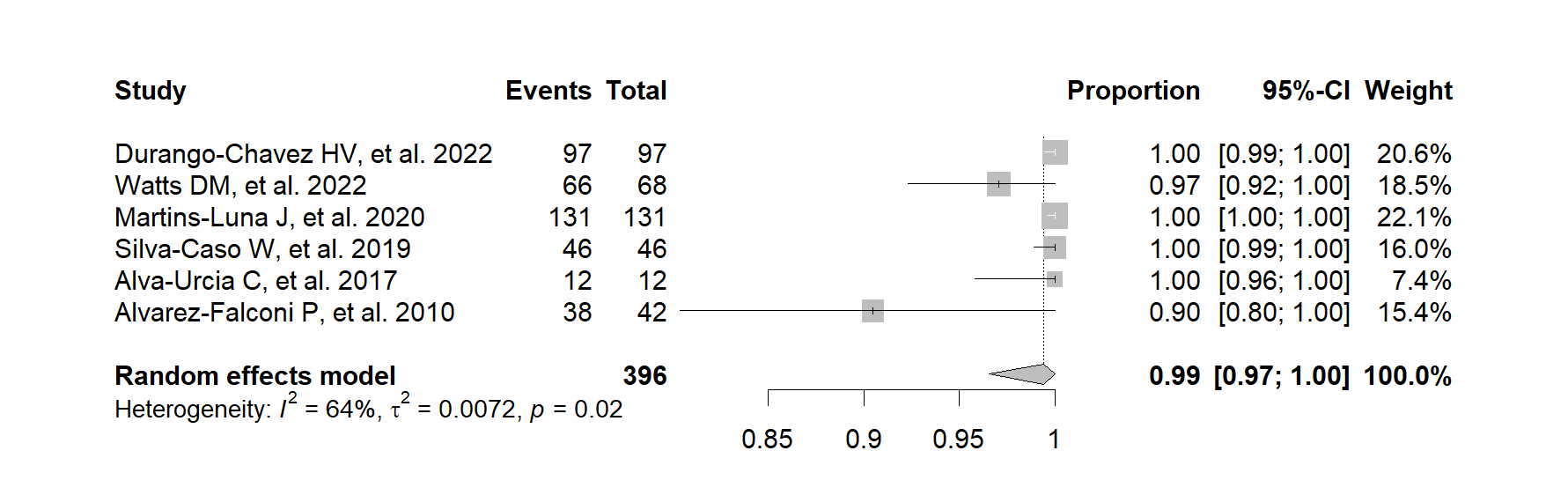

Supplement: S1 Fig — (TIF) [file pone.0337522.s008.tif]

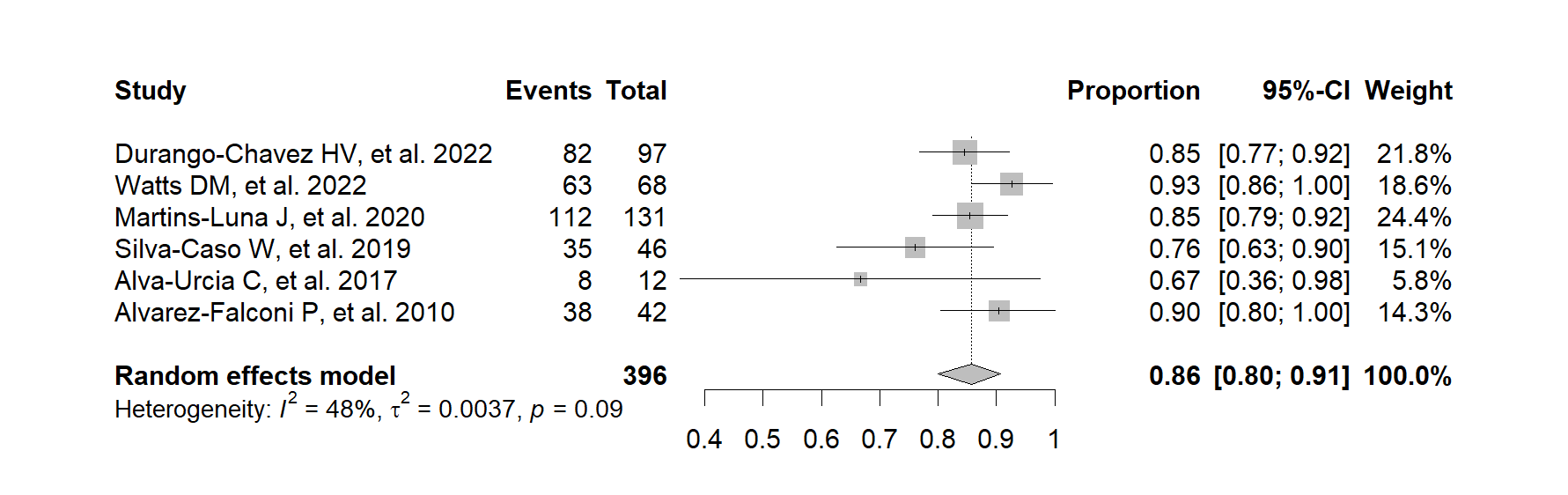

Supplement: S2 Fig — (TIF) [file pone.0337522.s009.tif]

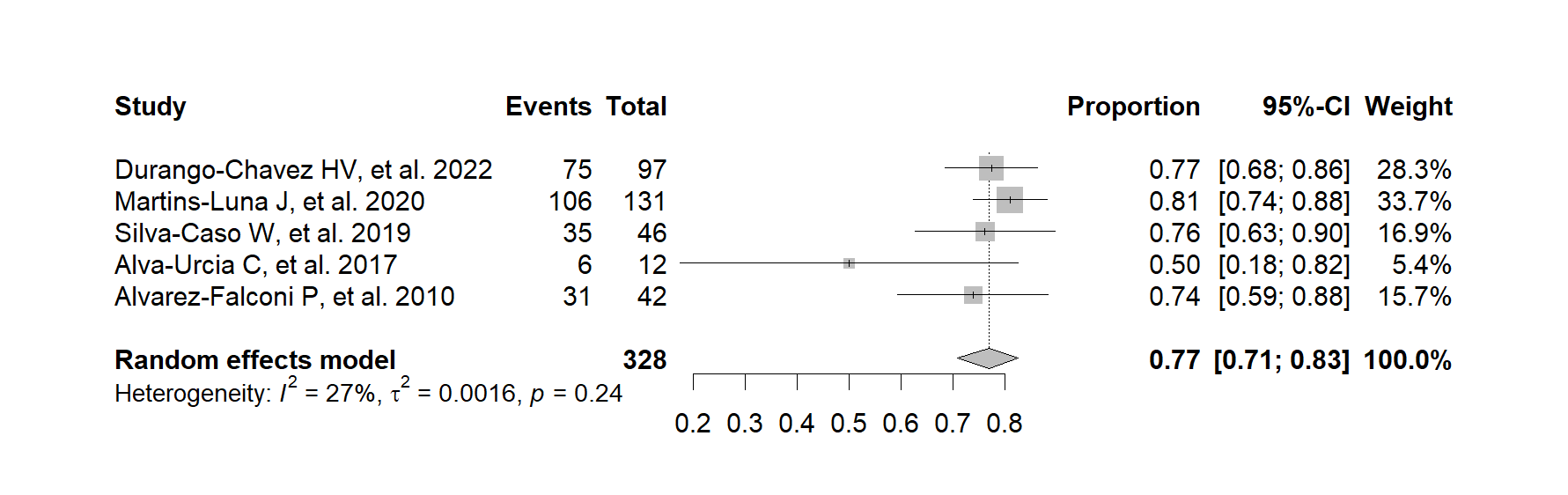

Supplement: S3 Fig — (TIF) [file pone.0337522.s010.tif]

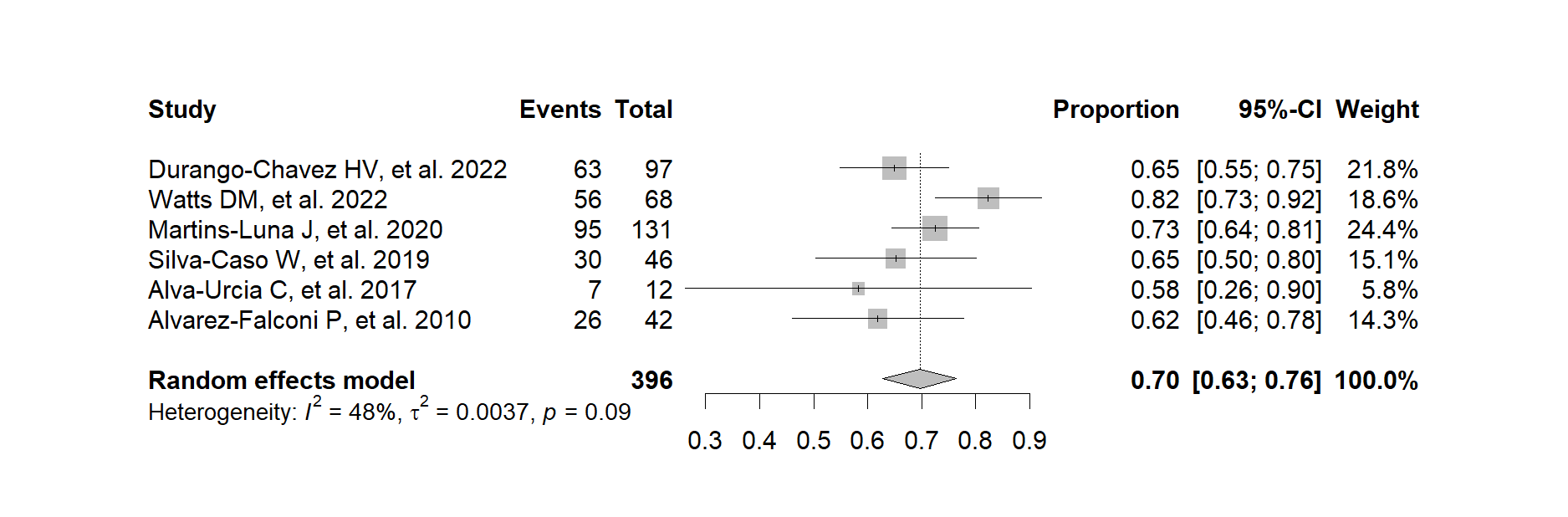

Supplement: S4 Fig — (TIF) [file pone.0337522.s011.tif]

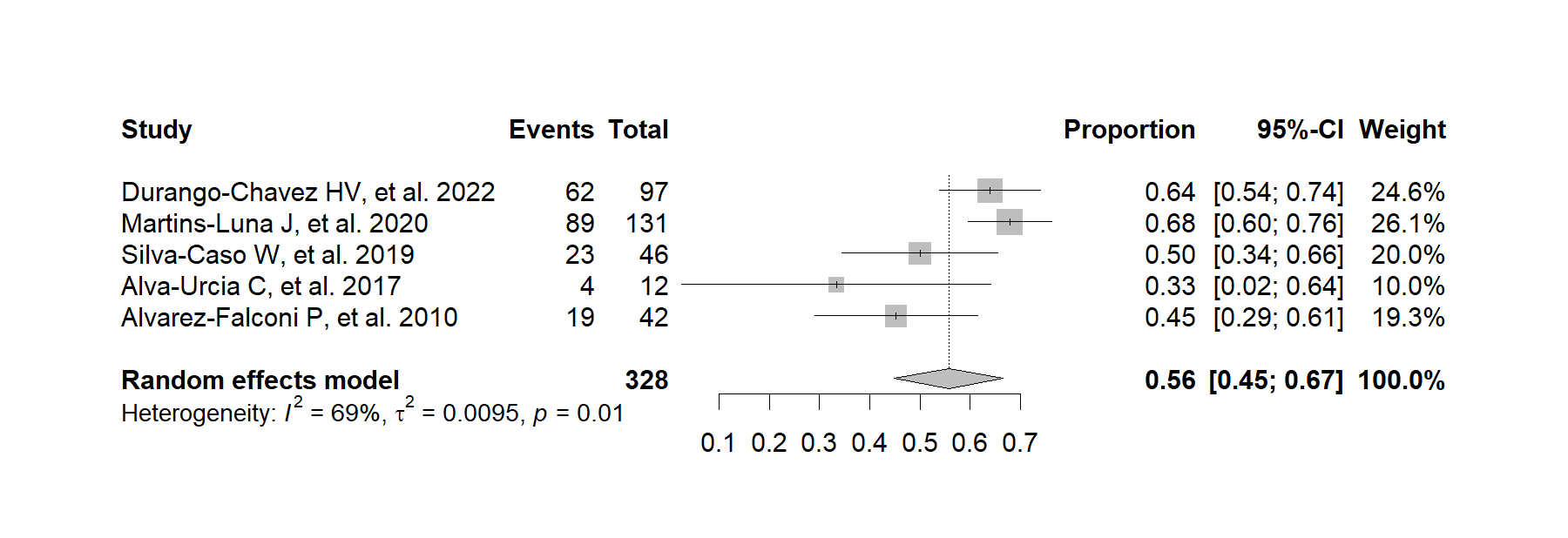

Supplement: S5 Fig — (TIF) [file pone.0337522.s012.tif]

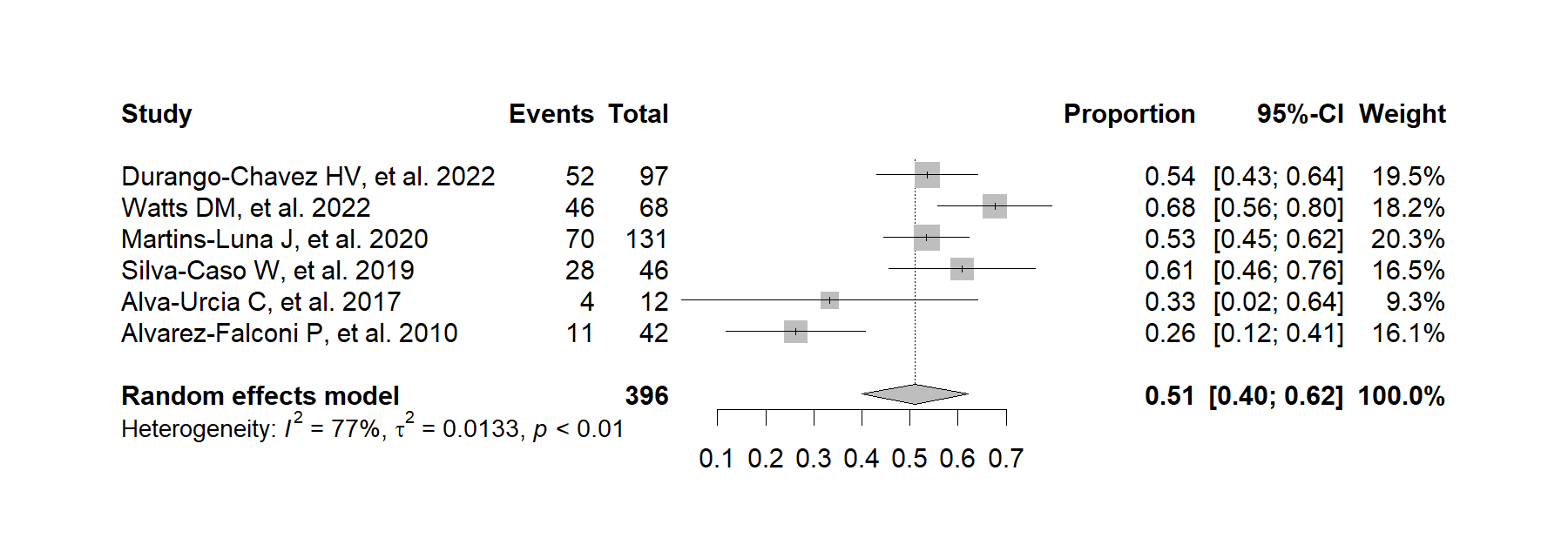

Supplement: S6 Fig — (TIF) [file pone.0337522.s013.tif]

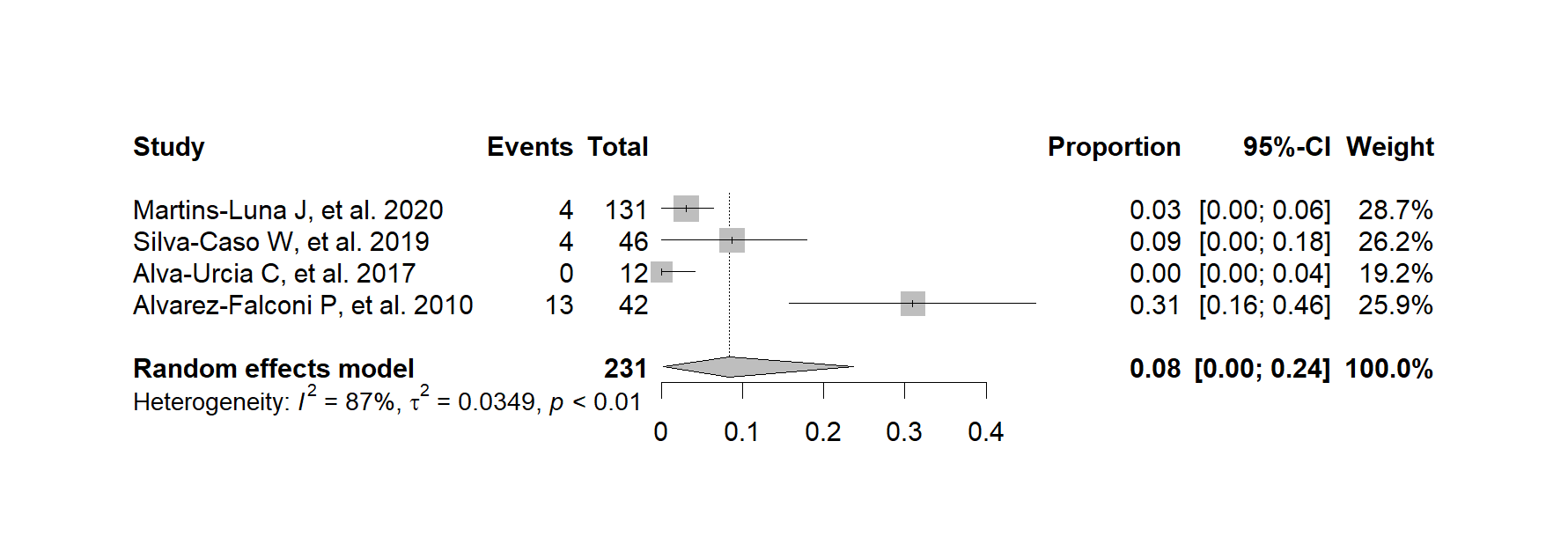

Supplement: S7 Fig — (TIF) [file pone.0337522.s014.tif]

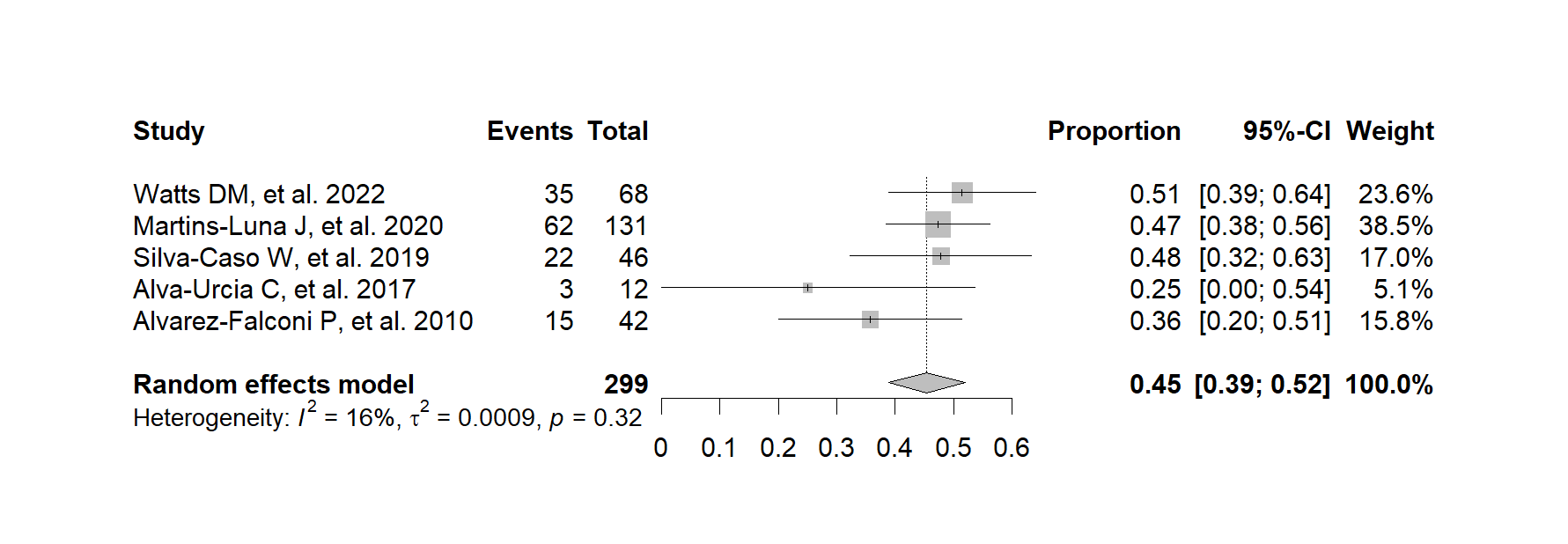

Supplement: S8 Fig — (TIF) [file pone.0337522.s015.tif]

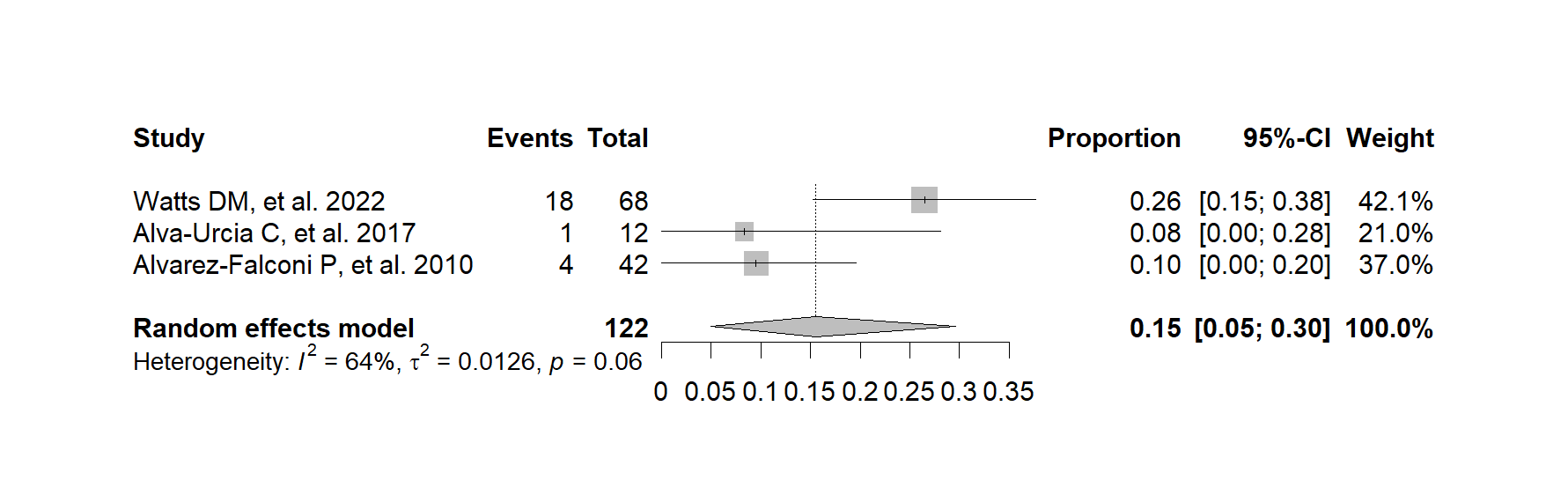

Supplement: S9 Fig — (TIF) [file pone.0337522.s016.tif]

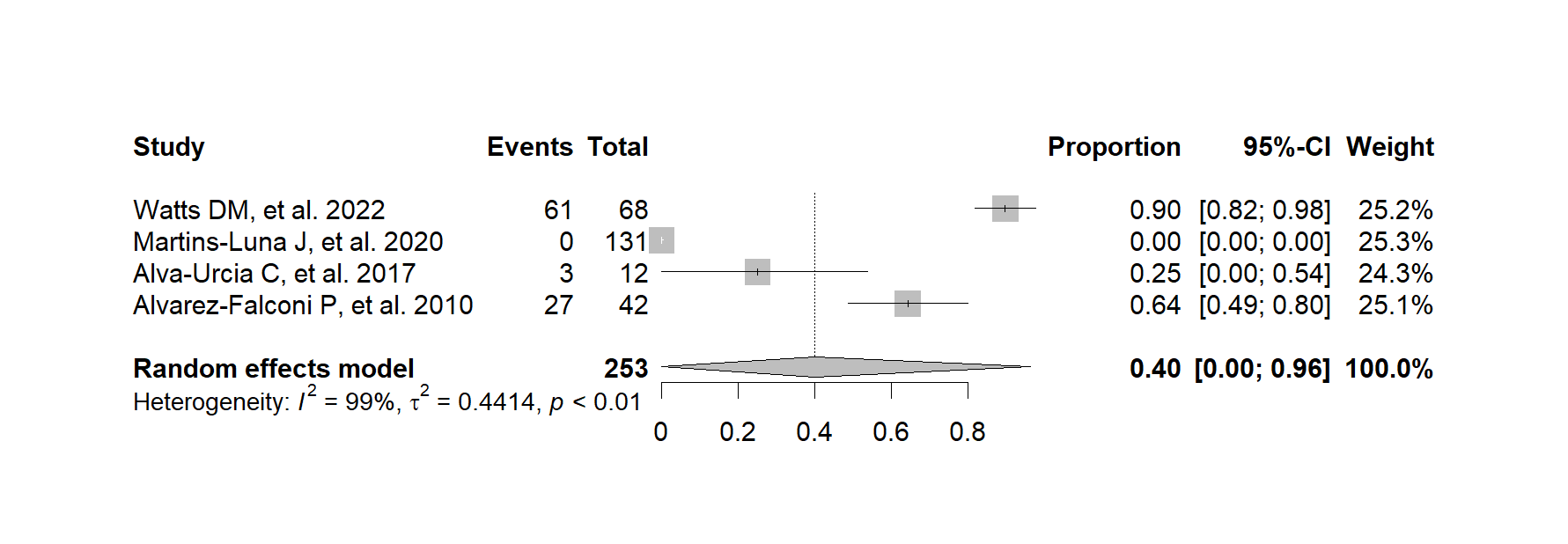

Supplement: S10 Fig — (TIF) [file pone.0337522.s017.tif]

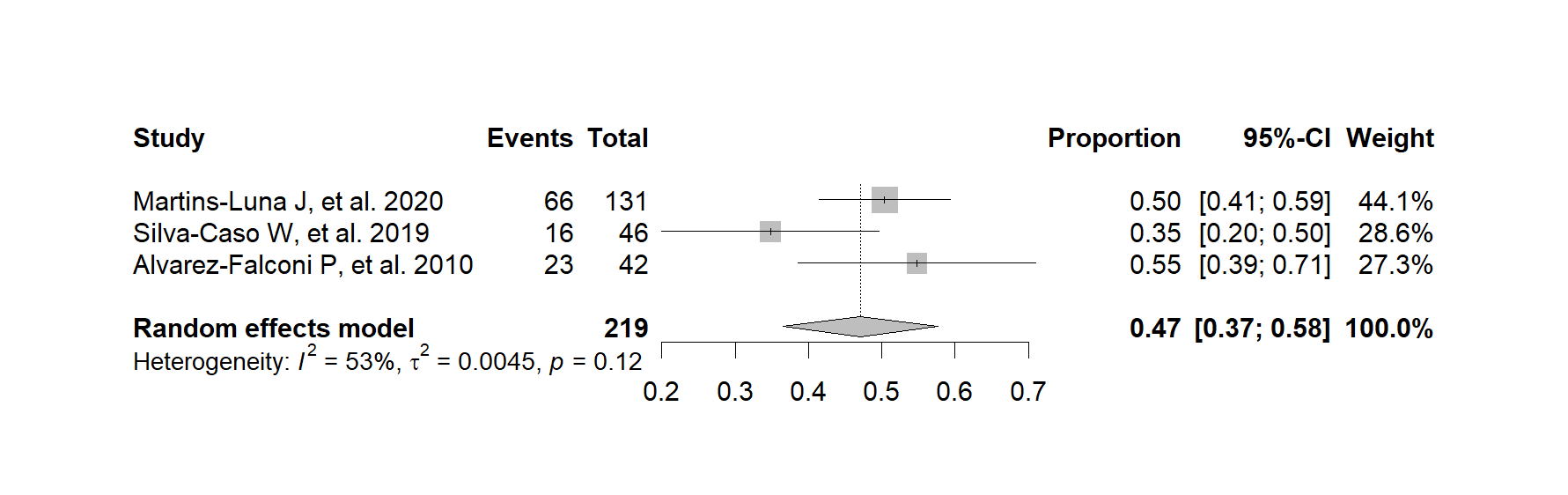

Supplement: S11 Fig — (TIF) [file pone.0337522.s018.tif]

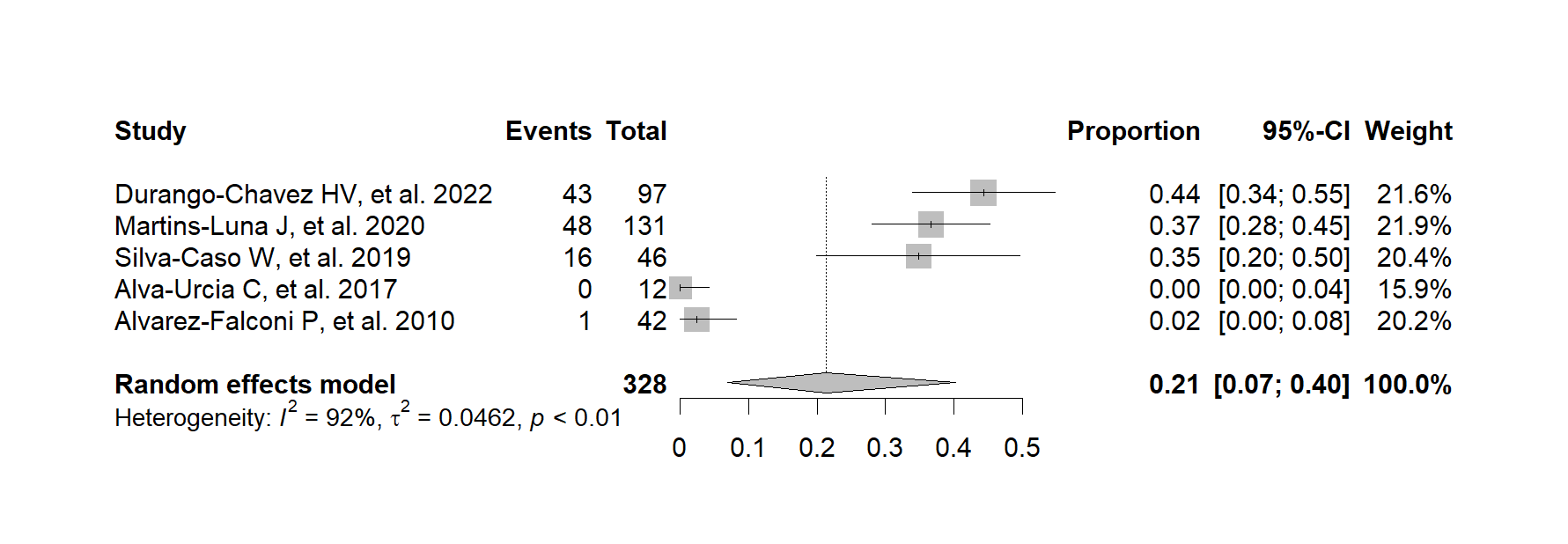

Supplement: S12 Fig — (TIF) [file pone.0337522.s019.tif]

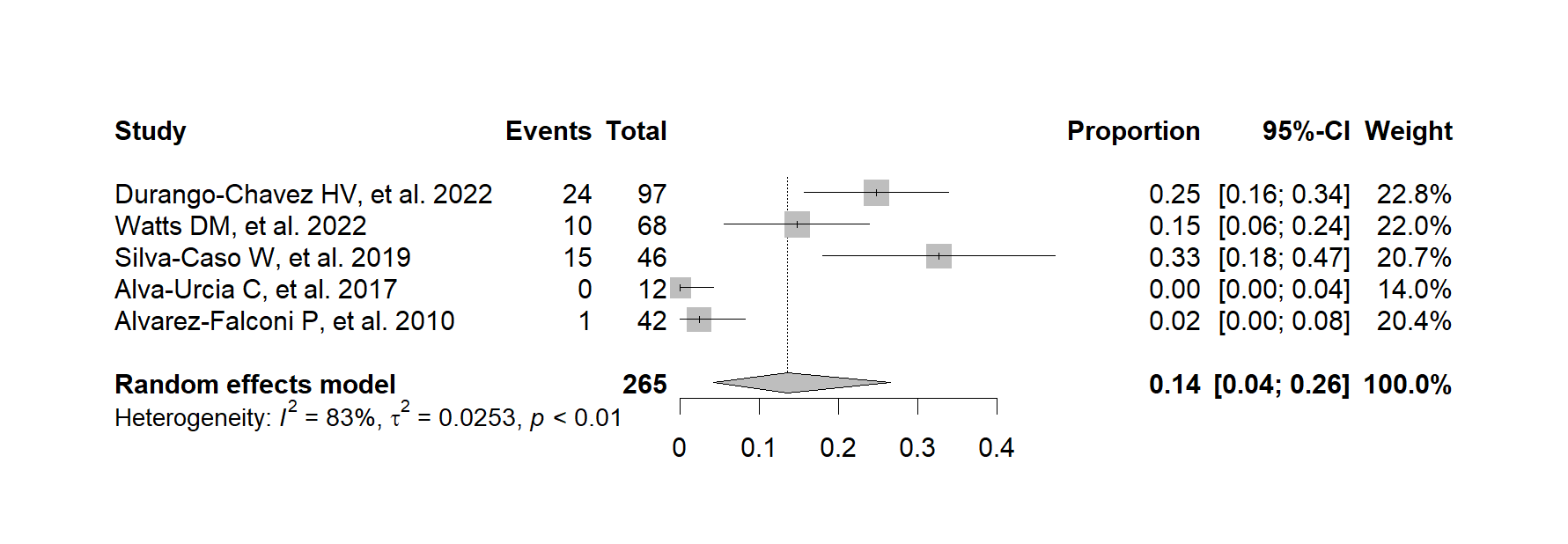

Supplement: S13 Fig — (TIF) [file pone.0337522.s020.tif]

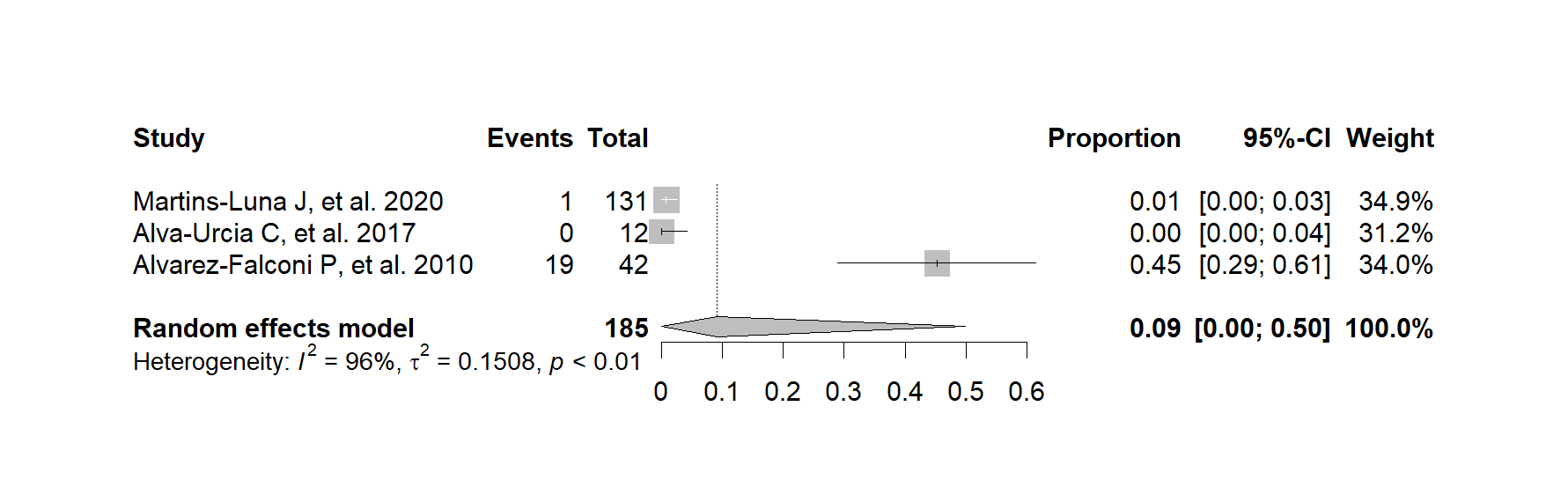

Supplement: S14 Fig — (TIF) [file pone.0337522.s021.tif]

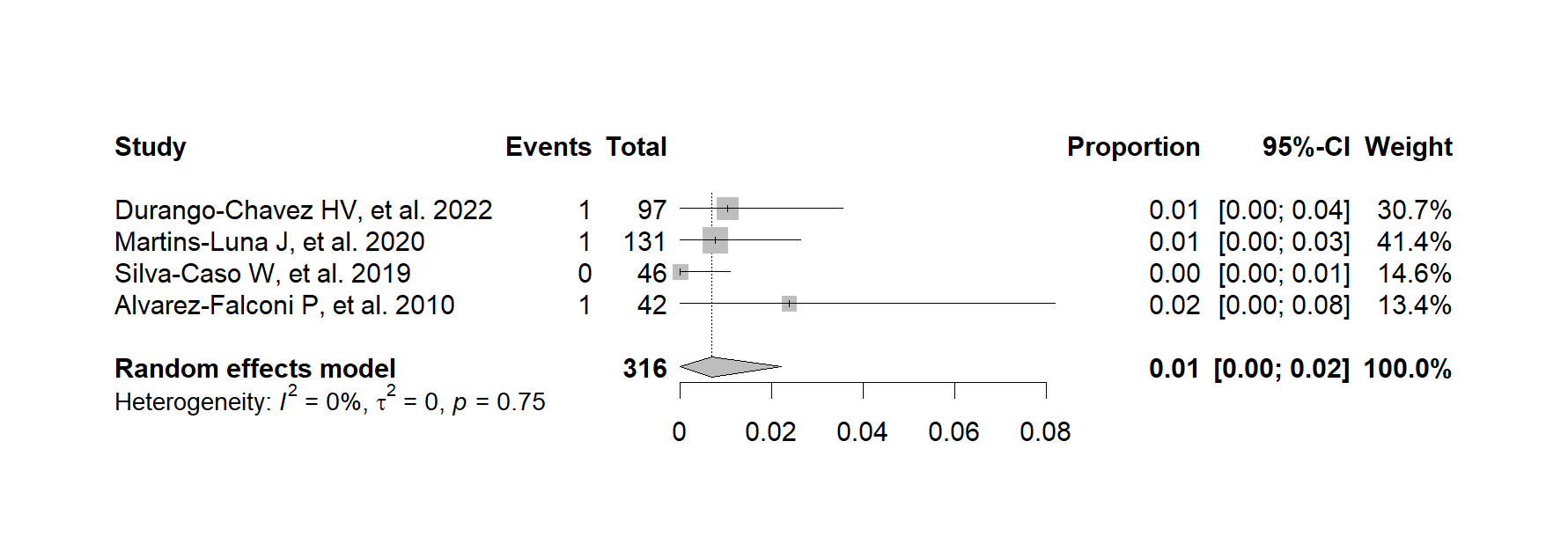

Supplement: S15 Fig — (TIF) [file pone.0337522.s022.tif]

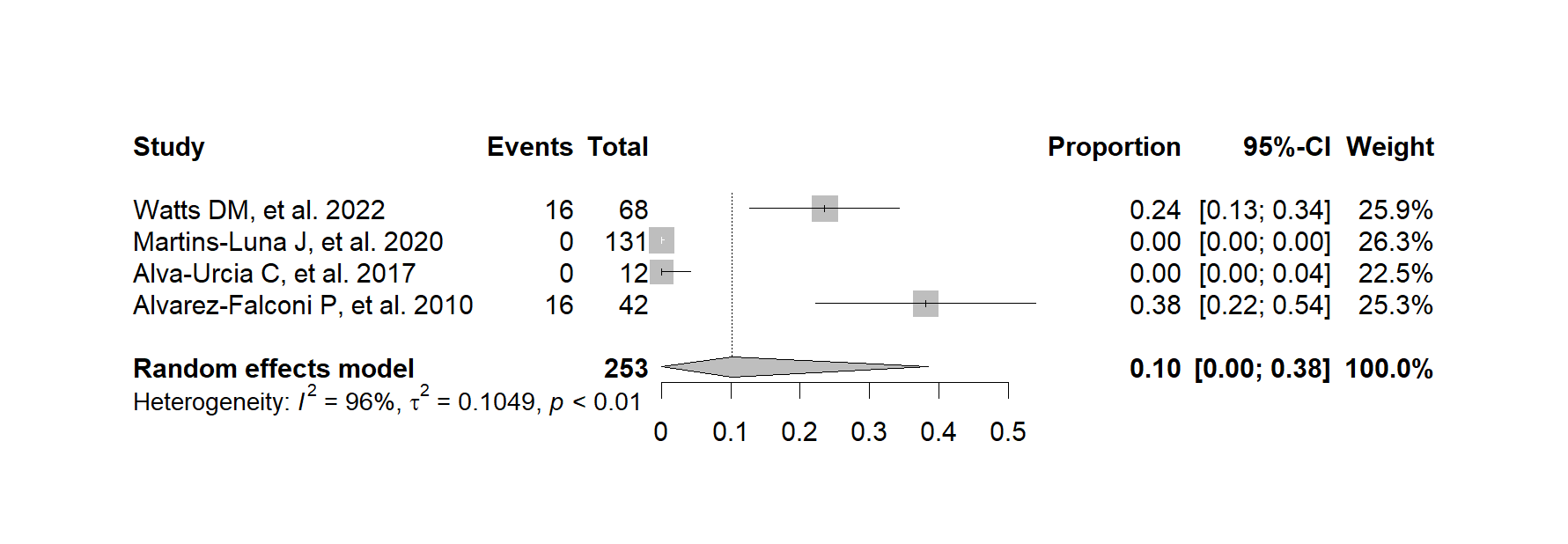

Supplement: S16 Fig — (TIF) [file pone.0337522.s023.tif]
