# Supplementary material for: Oropouche infection in Peruvian patients: A systematic review and meta-analysis
Source: PLoS One. 2025 Dec 4;20(12):e0337522. doi: 10.1371/journal.pone.0337522 (PMC12677477; doi:10.1371/journal.pone.0337522)
Supplement: Attachment — Submitted filename: Response to Editor.pdf [file pone.0337522.s024.pdf]

Editor's comment

1. We note that this manuscript is a systematic review or meta-analysis; our author guidelines therefore require that you use PRISMA guidance to help improve reporting quality of this type of study. Please upload copies of the completed PRISMA checklist as supporting information and a flowchart as Figure 1.

Information about the PRISMA guidance, and blank flowcharts and checklists, can be found here: http://www.prisma-statement.org/. Please note that if your study analyes clinical trials, we cannot proceed with consideration of your article until we have this information. If your meta-analysis or systematic review looks at studies other than clinical trials, then we request that you complete all parts of the PRISMA flow chart and checklist that are applicable. If you feel that this requirement should not apply to your study, please briefly explain the reason for this.

Response to the editor

Dear Editor, Thank you for your observation. We appreciate your attention to detail and the importance of adhering to PRISMA guidelines to enhance the reporting quality of systematic reviews and meta-analyses. As mentioned previously, our team has extensive experience in conducting systematic reviews, and we ensure that best practices are followed in the design of this study.

Regarding the PRISMA checklist, I confirm that it is included in the supplementary material file, as indicated in our previous response. However, I am attaching the document once again for your convenience.

I remain available for any further instructions and thank you in advance for your time and consideration.

2. Thank you for submitting the above manuscript to PLOS One. During our internal evaluation of the manuscript, we found significant text overlap between your submission and the following previously published works, some of which you are an author.

https://www.scielosp.org/article/rpsp/2024.v48/e136/

https://pubmed.ncbi.nlm.nih.gov/40561075/

We would like to make you aware that copying extracts from previous publications, especially outside the methods section, word-for-word is unacceptable. In addition, the reproduction of text from published reports has implications for the copyright that may apply to the publications.

Therefore, we cannot consider your manuscript as it stands. Please revise the manuscript to rephrase the duplicated text and fully cite all your sources, where appropriate.

Response to the editor

Dear Editor, thank you for your observations. First, I would like to clarify that our team is currently working on systematic reviews focused on determining the characteristics of patients diagnosed with prevalent infectious diseases in Peru.

Regarding the article you mentioned ("Epidemiological and clinical characteristics of Peruvian patients with mpox: A systematic review and meta-analysis"), it is an earlier version that served as a foundation for the current study in terms of methodology and outcome focus. As such, some text may overlap, as we followed a similar approach regarding structure and objectives. I have rephrased the text in the relevant sections and properly cited the published articles to ensure that readers and researchers understand that our research is built upon prior examples in order to improve methodological quality and facilitate comparison with other studies.

Regarding the observation about potential text overlap from previous publications, particularly outside the methods section, we have thoroughly reviewed the manuscript using Turnitin and rechecked the entire text. The publication by Tortosa et al. ("Key clinical manifestations to differentiate Oropouche fever from dengue and other arboviral diseases: a living systematic review") presents a different focus than ours, as it evaluates the frequency of symptoms in patients with Oropouche fever and compares them with those observed in dengue and other arboviral diseases. Moreover, we identified that some studies included in their review were not adequately evaluated, such as the one by Castillo Oré et al., which does not specifically analyze the Oropouche virus. This highlights a key difference with our focus. Our systematic review specifically targets studies conducted in Peru, which justifies our approach.

Lastly, we have appropriately cited all relevant sources in the methodology, introduction, and discussion sections to ensure proper referencing of prior works. We hope that these adjustments satisfactorily address all the observations.

I remain available for any further comments and appreciate your time and consideration.

Mario J. Valladares-Garrido

Escuela de Medicina Humana, Universidad Señor de Sipán, Chiclayo, Peru; vgarrido@uss.edu.pe (M.J.V.G)
