# Supplementary material for: Oropouche infection in Peruvian patients: A systematic review and meta-analysis
Source: PLoS One. 2025 Dec 4;20(12):e0337522. doi: 10.1371/journal.pone.0337522 (PMC12677477; doi:10.1371/journal.pone.0337522)
Supplement: Attachment — Submitted filename: Response to Reviewers.pdf [file pone.0337522.s025.pdf]

Dear **Editor**,

Thank you very much for reviewing our article, " ***Oropouche infection in Peruvian patients: a systematic review and meta-analysis***". Your suggestions and comments will be addressed below. Thank you for your valuable time and excellent review.

Editor's comments

1. The authors need to answer the comments of the reviewers and clarify the role of the vector in Oropouche virus transmission and the possibility of confusion between infection of DENV-3 and OROV symptoms.
   Our response: We sincerely appreciate your valuable feedback. In response to your request, we have expanded both the introduction and discussion sections regarding the role of vectors in the transmission of Oropouche virus (OROV). In addition to *Culicoides paraensis*, we now also mention *Aedes aegypti*, *Aedes albopictus*, and *Culex quinquefasciatus*, all of which play a role in the spread of the virus, particularly in urban areas. Furthermore, we've addressed the potential confusion between the symptoms of Oropouche virus and those of dengue virus serotypes, highlighting clinical similarities such as fever, headache, and myalgia, which make differential diagnosis challenging, especially in regions where both viruses co-circulate. We also added an analysis of how the introduction of DENV-3 has affected the clinical presentation of Oropouche fever, emphasizing the differences between DENV serotypes and their clinical impact, particularly in countries like Peru, where DENV-2 is more prevalent. We hope these revisions help clarify the concerns raised and improve the quality of the manuscript.

   Summary of the changes made:

   a. ***Modification of the introduction and discussion according to the reviewers' suggestions:***
   Significant adjustments were made to the introduction and discussion sections to appropriately incorporate and reflect the recommendations provided by the reviewers. These modifications aim to enhance the clarity and depth of the arguments presented, ensuring that key points are addressed with greater precision and coherence.
   b. ***Updating of the epidemiological data on OROV in Peru:***
   The epidemiological data related to Oropouche fever (OROV) in Peru were updated, incorporating the most recent figures and studies available. This provides a more accurate and up-to-date view of the epidemiological context of the disease in the country, enhancing the relevance and applicability of the findings in the local setting.
   c. ***Inclusion of a figure highlighting the circulation of arboviral diseases such as Oropouche, dengue, Zika, and chikungunya:***
   A figure was added illustrating the circulation of the main arboviral diseases in the region, including Oropouche, dengue, Zika, and chikungunya. This visualization allows for a quicker and clearer understanding of the distribution and interrelation of these diseases, facilitating data interpretation and the comparison of epidemiological trends.
   d. ***Review and standardization of acronyms throughout the article:***
   A thorough review was conducted to standardize the use of acronyms and abbreviations throughout the document, ensuring that each term was used

consistently. This enhances the readability and coherence of the article, preventing confusion and ensuring that readers can correctly interpret the abbreviations used.

e. Development of recommendations for future studies:

Specific recommendations for future research were added, based on the findings of the current work. These suggestions aim to guide further investigations that could expand knowledge on Oropouche fever and other arboviral diseases, as well as explore new areas of interest that may be relevant for advancing the prevention and control of these diseases.

f. Verification and correction of duplicated references:

A thorough process of verifying the bibliographic references was conducted, removing any duplicates and ensuring that all citations were correct and relevant. This review guarantees the integrity of the article, eliminating redundancies and improving the quality of the cited sources, thereby strengthening the credibility of the work.

Reviewer #1:

1. **Reviewer says:** The manuscript by León-Figueroa and colleagues addresses an increasingly worrisome pathogen from South American. The manuscript is exceptionally well written and uses standard techniques for reviewing the literature, assessing quality of relevant reports and performing statistical evaluations. I view this manuscript as a valuable addition to the literature on OROV and do not have substantive critisms. I do have two rather trivial editorial suggestions:

   Our response: We deeply appreciate your valuable comments and observations on our manuscript. Your recommendations have not only been essential in improving the quality of the information presented but have also strengthened our research, providing clarity and precision to the points discussed. We are pleased to know that the manuscript has been well received, and we will continue to work diligently to ensure that it meets the highest standards of quality.

2. **Reviewer says:** Line 43: Oropouche virus (OROV) -> OROV (you already defined OROV on line 33)

   Our response: The term OROV was corrected and reviewed throughout the article, ensuring consistency.

3. **Reviewer says:** Lines 56-60: I would suggest you delete all the %, CI and I^2 values from the abstract since they are presented in Table 2, and simply state "The most common symptoms were fever, headache, …" to enhance readability of the abstract.

   Our response: The suggested values were removed from the abstract, which has significantly improved its readability.

Reviewer #2:

Thank you for your valuable feedback and for providing constructive comments that have significantly improved the quality and clarity of our manuscript. Below is a detailed explanation of how we have addressed the observations provided:

1. **Reviewer says:** A more detailed description of the overlap between vectors and cases should be provided.

   Our response: We have improved the introduction and discussion sections to provide a more comprehensive understanding of the vectors involved in Oropouche virus (OROV) transmission. Specifically, we have expanded on the diversity of vectors transmitting OROV, including not only Culicoides paraensis but also Aedes aegypti, *Aedes albopictus*, and *Culex quinquefasciatus*, which are becoming increasingly relevant in urban settings with high population density.

   In response to the reviewer's comments regarding the overlap between vectors, we have incorporated recent studies that highlight the co-circulation of OROV with other arboviruses such as dengue. These studies suggest that the presence of multiple vectors, such as Aedes and Culicoides species, increases the complexity of transmission dynamics, potentially leading to higher morbidity and complications in diagnosis.

   Additionally, we have clarified the impact of environmental factors on vector populations, noting that rising temperatures and changing precipitation patterns have created more favorable breeding conditions for Culicoides mosquitoes. Recent studies have shown how climate change can affect the geographical spread of these vectors, further increasing the risk of OROV outbreaks.

2. **Reviewer says:** It is also necessary to address the changes in symptoms observed when DENV-3 was introduced into the region.
   Our response: We agree with the reviewer’s suggestion to address the potential confusion between the symptoms of Oropouche virus (OROV) infections and dengue virus, particularly in regions where both diseases are endemic. In the manuscript, we have added a section that discusses the overlapping clinical manifestations of OROV and dengue, such as fever, headache, and myalgia. These similarities make differential diagnosis challenging, especially in areas with high co-circulation of multiple mosquito-borne viruses.

   Additionally, we have incorporated recent findings that show how the introduction of DENV-3 in areas affected by OROV has influenced the clinical presentation of Oropouche fever. However, the discussion of dengue and other arboviruses, such as Zika and Chikungunya, has been addressed in a general way. In Peru, DENV-2 is more common, and when it occurs as a secondary infection,

it tends to be more lethal with a worse prognosis. On the other hand, DENV-3, in studies where it occurs as a primary infection, is associated with a higher risk of complications.

3. **Reviewer says:** The data are valuable, but they need to be presented in a more accessible and reader-friendly manner.
   Our response: To enhance the manuscript and address the reviewer's request for a more accessible and reader-friendly presentation, we have added new references from recent studies that explore the interaction between OROV and other arboviruses, the role of climate change in vector distribution, and the diagnostic challenges posed by co-infections. These references not only support our discussion but also provide additional context for understanding the evolving epidemiology of OROV.

If you have any comments or recommendations, we are ready to respond.

Sincerely,

Mario J. Valladares-Garrido

Escuela de Medicina Humana, Universidad Señor de Sipán, Chiclayo, Peru; vgarrido@uss.edu.pe
