# Supplementary material for: Oropouche infection in Peruvian patients: A systematic review and meta-analysis
Source: PLoS One. 2025 Dec 4;20(12):e0337522. doi: 10.1371/journal.pone.0337522 (PMC12677477; doi:10.1371/journal.pone.0337522)
Supplement: Attachment — Submitted filename: Response_to_Reviewers_auresp_2.pdf [file pone.0337522.s026.pdf]

Dear **Editor**,

Thank you very much for reviewing our article, " ***Oropouche infection in Peruvian patients: a systematic review and meta-analysis***". Your suggestions and comments will be addressed below. Thank you for your valuable time and excellent review.

Editor's comments

1. To proceed with publication, I would appreciate it if you would take into account the reviewers' recent comments.
   Our response:
   We have carefully reviewed their suggestions and made the necessary revisions to improve the manuscript. We hope these changes address their concerns and that the manuscript is now ready for publication.

Reviewer #1:

1. **Reviewer says:** The changes you made certainly enhance the clarity of several aspects of the manuscript. Very nice job.

   Our response: Thank you very much for your review. Your comments and suggestions have been crucial in improving the quality of our article.

Reviewer #3:

1. The manuscript "Oropouche infection in Peruvian patients: a systematic review and meta-analysis." presents an interesting analysis of the epidemiological profile of OROV in Peru. I have some comments and suggestions before making a final decision.

   Our response: Thank you for your thoughtful review and for recognizing the value of our analysis. We appreciate your comments and suggestions, and we will carefully address them to further improve the manuscript. We look forward to making the necessary revisions and submitting an updated version for your final consideration.

   -> Introduction

2. **Reviewer says:** Highlight deaths from OROV in Brazil. This finding is unprecedented.
   Our response: Thank you very much for your valuable comments and suggestions. I have made the necessary revisions to highlight the fatal cases of Oropouche virus (OROV) in Brazil, as requested. In the revised version, I have emphasized the unprecedented finding of these deaths, marking a significant increase in the severity of the virus's impact in non-endemic regions such as Brazil. This observation has been included to underline the urgent need for attention due to the potential evolution of more virulent strains.

Thank you again for your insightful feedback, which has been very helpful in improving the clarity and precision of the manuscript.

3. **Reviewer says:** Regarding the differential diagnosis between dengue and Oropouche, this is a very important issue and some studies have been dedicated to this topic. I suggest reading and mentioning some, such as doi:10.1016/j.lana.2024.100718. e doi: 10.1186/s12985-025-02945-x.

   Our response: Thank you for the valuable recommendation. I have incorporated the suggested information, highlighting the urgent need to strengthen healthcare personnel training in vulnerable areas where the simultaneous circulation of various arboviruses is common. Additionally, I have emphasized that the overlap in symptoms between diseases such as dengue and Oropouche further complicates accurate diagnosis, increasing the risk of misdiagnosis and underdiagnosis. In this context, given the increasing spread of Oropouche alongside other arboviruses, I believe it is essential to implement more refined diagnostic protocols and improve surveillance systems to effectively differentiate these diseases and ensure timely and accurate medical responses.

4. **Reviewer says:** There is a multifactorial discussion about the increase in OROV cases/circulation. In addition to environmental issues, some authors have discussed important changes in the viral genome. I suggest reading and citing some studies, such as doi: 10.1038/s41591-024-03300-3, doi: 10.1101/2024.07.27.24310296 e doi: 10.1002/jmv.70112.

   Our response: Thank you for your observation. I have reviewed your comment, and I agree that the discussion regarding the increase in OROV cases should address both environmental factors and potential genetic changes in the virus. In my text, I included information about a new reassortant OROV strain that has emerged in the Brazilian Amazon region, which combines genetic segments from strains in the eastern Amazon and neighboring countries such as Peru, Colombia, and Ecuador. This strain has shown higher replication and virulence, contributing to a reduction in the effectiveness of pre-existing immunity in previously infected individuals. Additionally, I highlighted the role of human migration and the movement of vectors as facilitating factors for the virus's spread in the region, which is also considered in recent literature.

5. **Reviewer says:** Figure 1: Write the viral taxa in italics; dengue and chikungunya do not start with a capital letter; clarify what the numbers after the virus names mean (confirmed cases?); to what time interval do the cases correspond?.

   Our response: Thank you very much for your valuable suggestions. The corrections have been made to Figure 1, including the italicization of the viral taxa names, the correction of the disease names with a lowercase initial letter, and the clarification that the numbers represent confirmed cases, with the corresponding time interval specified (Epidemiological Week 38 of 2025).
   Once again, thank you for your feedback, and I remain available for any further comments.

6. **Reviewer says:** I don't understand why the manuscript's strengths and limitations are referenced and why they are the same references -> This study presents several strengths [10,32,33]; The study has several key limitations [10,32,33].

   Our response: Thank you for your observation. We have removed the references from the indicated sections. The context arose because these references pertained to other meta-analyses that shared common strengths and limitations. However, we have made the correction as per your suggestion

If you have any comments or recommendations, we are ready to respond.

Sincerely,

Mario J. Valladares-Garrido

Escuela de Medicina Humana, Universidad Señor de Sipán, Chiclayo, Peru; vgarrido@uss.edu.pe
